# Moebius sequence –a multidisciplinary clinical approach

**DOI:** 10.1186/s13023-016-0559-z

**Published:** 2017-01-06

**Authors:** Line Kjeldgaard Pedersen, Rikke Damkjær Maimburg, Jens Michael Hertz, Hans Gjørup, Thomas Klit Pedersen, Bjarne Møller-Madsen, John Rosendahl Østergaard

**Affiliations:** 1 Department of Clinical Medicine, Aarhus University Hospital, Aarhus, Denmark; 2 Department of Childrens Orthopaedics, Aarhus University Hospital, Aarhus, Denmark; 3 Department of Gynaecology-Obstetrics, Aarhus University Hospital, Aarhus, Denmark; 4 Department of Clinical Genetics, Odense University Hospital, Odense, Denmark; 5 Department of Maxillofacial Surgery, Aarhus University Hospital, Aarhus, Denmark; 6 Department of Orthodontics, Aarhus University, Aarhus, Denmark; 7 Center for Rare Diseases, Department of Pediatrics, Aarhus University Hospital, Aarhus, Denmark

**Keywords:** Moebius Sequence, Rare Disease, Children, Multidisciplinary Approach

## Abstract

**Background:**

Moebius Sequence (MS) is a rare disorder defined by bilateral congenital paralysis of the abducens and facial nerves in combination with various odontological, craniofacial, ophthalmological and orthopaedic conditions. The aetiology is still unknown; but both genetic (de novo mutations) and vascular events in utero are reported. The purpose of present study was through a multidisciplinary clinical approach to examine children diagnosed with Moebius-like symptoms. Ten children underwent odontological, ophthalmological, obstetric, paediatric, orthopaedic, genetic, radiological and photographical evaluation. Five patients maintained the diagnosis of MS according to the diagnostic criteria.

**Results:**

All five patients had bilateral facial and abducens paralysis confirmed by ophthalmological examination. Three of five had normal brain MR imaging. Two had missing facial nerves and one had missing abducens nerves. The Strengths and Difficulties Questionnaire (SDQ) showed normal scores in three of five patients. Interestingly, two of five children were born to mothers with uterine abnormalities (unicornuate/bicornuate uterus). In the odontological examination three of five showed enamel hypomineralisation. All five had abnormal orofacial motor function and maxillary prognathism. Two patients had adactyly, syndactyly and brachydactyly. None of the five patients had Poland anomaly, hip dislocation or dysplasia but all had a mild degree of scoliosis. We observed congenital club-feet, calcaneovalgus deformities, macrodactyly of one or more toes or curly toes. Pedobarography showed plantar pressures within normal ranges.

**Conclusions:**

Adherence to standard diagnostic criteria is central in the diagnosis of MS. An accurate diagnosis is the basis for correct discussion of other relevant concomitant symptoms of MS, genetic testing and evaluation of prognosis. The multidisciplinary approach and adherence to diagnostic criteria taken in present study increases the knowledge on the relationship between genotype, phenotype and symptomatology of MS.

## Background

Moebius Sequence (MS) (OMIM 157900), previously known as Moebius syndrome, is a rare disorder defined by congenital paralysis of the 6^th^ and 7^th^ cranial nerves [1, 2]. The disorder is congenital with chronic consequenses and is previously diagnosed after birth or during early infancy by the child’s facial features. Paralysis of other cranial nerves, malformations of orofacial structures, and limb anomalies may also be present [3]. The incidence of MS varies from 0.00002 to 0.002% [4], with a sex ratio of 1:1 [5], although a Swedish study found a female-male-ratio of 1:3 and a high occurrence of misdiagnosis. The term *sequence* is preferred to *syndrome* since it defines a cascade of secondary events after an initial insult during the embryonic development [6, 7] in addition to a possible genetic aetiology [8]. A large heterogenic group of syndromes with variable symptoms of uni- or bilateral facial and abducens paralysis in conjunction with a range of other systemic anomalies have been described. Different opinions on how to differentiate between these conditions have resulted in varying diagnostic criteria for MS and consequently a bias in published case reports and studies on MS [7]. MS belongs to a large group of similar syndromes called “Oro-Mandibular-Limb hypogenesis syndrome” (OMLH) characterised by varying cranial nerve palsies and craniofacial and limb anomalies sometimes combined with aplasia of the pectoral muscle (Poland anomaly) among others Robin complex, Moebius-like syndrome, Carey-Fineman-Ziter Syndrome, Hanhart, hypoglossia-hypodactyly and glossopalatinus ankylosis [7, 9, 10].

The aetiology of MS is unknown. Most cases are sporadic but familial occurrence has been reported. Both genetic and non-genetic factors are believed to be significant for the development of MS. Non-genetic causes are primarily thought to involve vascular events with interruption or alteration of the blood supply of the embryo causing damage to cranial nerve centres leading to an abnormal positioning of the foetus causing unusual pressure in parts of the developing brain [11]. Secondary causes include exposure to teratogens in early pregnancy (benzodiazepines, misoprostols, thalidomide, cocaine, alcohol, hyperthermia, hypoxia and rubella) [3, 12]. Regarding the genetics factors of MS two different loci for MS at 3q21-q22 and 10q, respectively, have been reported [13, 14]. Recently, Tomas-Roca et al. [8] reported de novo mutations in MS patients affecting the *PLXND1* and *REV3L* genes which cause a defect in the facial branchiomotor neuron migration supporting these genes as causative for a proportion of MS cases.

Other concomitant symptoms in MS include paralysis of other cranial nerves, malformation of the orofacial skeleton and anomalies of the extremities, most often club-feet [5]. Oral manifestations may present as hypodontia, cleft palate, mandibular hypoplasia, abnormal tongue movements and incompetent lip closure [6, 15, 16]. Functional abnormalities of face and mouth have been reported: lack of facial expression, difficulties in speaking, eating, swallowing and restricted mouth opening [6, 17]. Comorbidities of MS include language difficulties, no ability to smile and a risk of a wrongful diagnosis of mental retardation. A study found that patients with MS had the same intelligence, memory and ability to concentrate as the background population. In addition almost all school-age children with MS below 17 years children were found to attend normal elementary or secondary schools indicating normal developmental milestones [18]. Other studies have reported a notably higher incidence of autism in patients with MS [19, 20].

MS is complex and entails lifelong treatment and knowledge of the disorder is sparse. A multidisciplinary clinically approach is necessary in order to optimize the diagnosis, treatment and advice given to these patients. In the present study, we invited all patients in the Western part of Denmark known to have MS or a Moebius-like Syndrome to a multidisciplinary evaluation including odontological, ophthalmological, obstetric, paediatric, orthopaedic, genetic, radiological and photographical evaluation.

## Methods

**Fig. 1:**
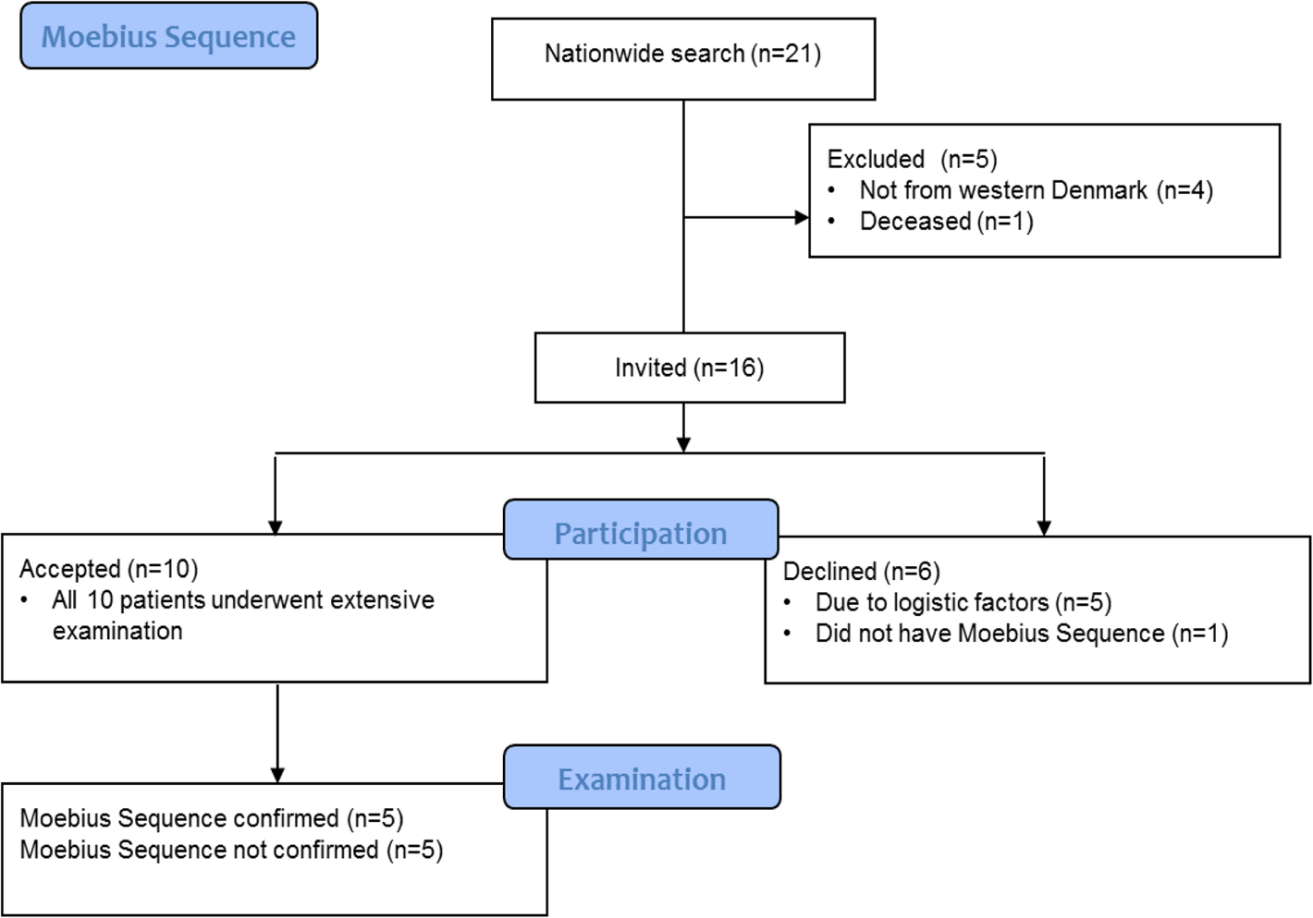
Consort 2010 Flow Diagram of the inclusion process

On initial search in the Danish National Patient Register, our clinical practices and the Danish Moebius Patients’ Association, 21 patients were identified and 16 patients from the western part of Denmark were invited to participate in the present study. The study was approved by the Central Denmark Region Committee on Biomedical Research Ethics no. 2010-41-5212 and the Danish Data Protection Agency. Six patients declined to participate due to logistic factors and ten patients accepted. Informed written consent from both parents was obtained. In total ten children were enrolled in this study at Aarhus University Hospital from 2012 to 2013. The inclusion process is presented in Fig. 1. In this study we define MS as congenital bilateral paralysis of the 6^th^ and 7^th^ cranial nerves.

A multidisciplinary set-up including paediatrics, ophthalmology, odontology, genetics, obstetrics, radiology and orthopaedics examined all patients. Data were obtained from interviews and questionnaires by patients and parents as well as from clinical examination of the patient including magnetic resonance imaging (MRI) of the brain. Cognitive aspects (development disorders, mental retardation and autism) and neuro-psychiatric aspects were assessed by a parent questionnaire (Strengths and Difficulties Questionnaires, SDQ) [21]. Furthermore medical records were reviewed.

Genetic evaluation included drawing up a pedigree for each family and collection of information on Moebius-like symptoms in relatives and parental consanguinity. Results from previous genetic tests were obtained from medical records, and the results of chromosome analysis from the Danish Cytogenetic Central Register.

Obstetric and neonatal evaluation included information on maternal exposure and complications in pregnancy and birth (in particular the foetal presentation, instrumental and active delivery methods e.g. McRobert manoeuvre, vacuum- and forceps delivery and caesarean section). Data were obtained on birth weight, birth length and head circumference, Apgar score, umbilical pH measure, nutritional problems (including use of stomach tube), icterus, hypoglycaemia, antibiotics and continuous positive airway pressure (CPAP) treatment.

Ophthalmological examinations included a full eye examination focusing on eye motility as well as full standard ophthalmological and orthoptic examination.

The odontological and craniofacial examination was clinical and radiological. The clinical examination included a visual assessment of dental anomalies, dental status (number of teeth, decayed, missed or filled surfaces due to caries (DMFS)), presence of dental erosion, dental occlusion (molar occlusion, horizontal overbite (HOB), and vertical overbite (VOB) in mm), gingival health (no. of incisors and first molars with bleeding on probing), and morphological characteristics of the tongue. In addition, the mouth opening capacity (MOC) (i.e., maximal interincisal distance on unassisted mouth opening + VOB) was compared to a reference value (the mean value in 20719 unselected children, 4–17 years) [22], and signs of temporomandibular dysfunction were assessed. The orofacial motor function was tested by The Nordic Orofacial Test- Screening (NOT-S) [23]. The radiological examination included a visual assessment of radiological signs on dental or craniofacial anomaly and pathology of the temporo-mandibular joint (TMJ). A cephalometric evaluation of the craniofacial morphology was performed according to a modification of the method by Bjørk and Solow [24].

Orthopaedic examinations included a complete history of orthopaedic complaints and previous surgery. Physical examination included visual evaluation of known deformities, range of motion testing, height and weight measurements. Assessments of limb hypoplasia, adactyly, syndactyly, camptodactyly, brachydactyly, Poland anomaly, scoliosis, leg length discrepancies, talipes equinovarus, pes calcaneovalgus, pes cavus, macrodactyly, curly toes, general malformations and hip dislocation were made. Four of five patients were examined with both static and dynamic pedobarography using the HRMAT Tekscan (Clin Seat Type 5315 Sensor, Tekscan, Boston, Mass, USA) to assess balance parameters as well as total and regional plantar pressure, area and force.

Clinical appearance was documented with clinical photographs of full body (back and front), hands (dorsal and ventral aspects), feet, standing (dorsal, anterior and posterior aspects), face (front, 45° right/left, 90° right/left), eyes (relaxed, closed, attempting to look right/left) and teeth (front, 45° right/left, upper, lower).

Radiological examinations included x-rays of the feet standing (dorsal and lateral aspects), hands (dorsal aspect), thorax (PA aspect), pelvis (PA aspect) and spine (PA, lateral). If the patient had undergone a recent radiographic examination the relevant view was omitted.

## Results

### Patients not meeting MS diagnostic criteria defined for this study

In five patients, the diagnosis of MS was withdrawn after examination by the multidisciplinary team since they did not have bilateral facial and abducens paralysis (Table 1).

**Table 1:** Patients not meeting MS diagnostic criteria defined for this study

	Facial paralysis	Abducens paralysis	Symptoms/findings	Diagnosis
No. 6	Unilateral	No	Hypermetropia, Achilles contracture	Unknown
No. 7	No	No	Fibrotic eye muscles, divergent strabismus, bilateral clubfeet, leukomalasia of the cerebrum with periventricular pathologies	Congenital Fibrosis of Extraocular Muscles type 3A (OMIM #600638) and Artrogryposis Multiplex Congenita
No. 8	No	No	Congenital vocal cord paralysis	Unknown
No. 9	Affected	Unilateral	Apraxia of the oculomotor nerves, midfacial hypoplasia, unilateral club-foot, anisomelia, Poland	Artrogryposis Multiplex Congenita.
No. 10	Unilateral	No	Congenital syringomelia, anal atresia, hypospadia, fibula atresia, cruciate ligament aplasia	Unknown

In this study we define MS as congenital bilateral paralysis of the facial and abducens nerves

### Genetic findings

After evaluation five patients maintained the diagnosis of MS. All five patients were isolated cases without a family history of Moebius-like symptoms, and without parental consanguinity. Normal karyotypes were registered for all five patients using standard chromosome analysis in Q-banding of cultured lymphocytes from peripheral blood.

### Obstetric findings

The obstetric outcomes for the children with a MS diagnosis were generally normal (Table 2). All children were conceived spontaneously. Four out of five children were born to nulliparous women at term. However, two children were born late preterm in week 35 and 36, respectively. Four out of five children were born vaginally in cephalic presentation. One child was delivered by cesarean section in breech presentation. Successfully versio externa in pregnancy was performed in patient no 4. Four out of five children had an Apgar score of 10 at five minutes. Breastfeeding was established in two out of five children during hospital stay. All mothers had good physical and mental health in the pre-pregnancy period as well as during pregnancy. However, it is noteworthy that two of the mothers had been diagnosed with uterine abnormalities; a unicornuate (patient no. 5) and bicornuate uterus (patient no. 4), respectively.

**Table 2:** Comparison of symptomatology in five patients with MS

	No. 1	No. 2	No. 3	No. 4	No. 5
Baseline demographics					
Age (yrs)	3	7	15	18	19
Sex (M: Male, F: Female)	M	F	F	F	F
Height (cm)	103	137	166.5	167	160
Weight (kg)	16.5	30.3	47.5	60	46
Mental and developmental					
Strengths and Difficulties Questionnaire (SDQ)					
Overall Stress (0–13)	8	2	3	14	13
Emotional distress (0–2)	1	0	1	6	4
Behavioural difficulties (0–3)	2	0	1	0	2
Hyperactivity and concentration difficulties (0–5)	4	0	1	4	5
Difficulties getting along with other children (0–2)	1	2	0	4	2
Kind and helpful behavior (7–10)	8	10	7	7	9
Impact of any difficulties on the child’s life (0)	0	0	0	4	1
Diagnostic predictions LR: Low Risk, MR: Medium Risk					
Any disorder	LR	LR	LR	MR	LR
Emotional disorder (anxiety, depression etc.)	LR	LR	LR	MR	LR
Behavioural disorder	LR	LR	LR	LR	LR
Hyperactivity or concentration disorder	LR	LR	LR	LR	LR
Obstetrics					
Conception (S:Spontaneous)	S	S	S	S	S
Mothers parity (0:nullipara, 1:multipara)	0	0	1	0	0
Mothers uterus	normal	normal	normal	bicornuate	unicornuate
Gestational age (weeks)	41	40	35	39	36
Birth weight (grams)	3240	3600	1950	3500	2135
Fetal presentation (C:cephalic, B: breach)	C	C	C	C	B
Delivery mode (V: vaginal, CS: caesarean section)	V	V	V	V	CS
Apgar Score (1 & 5 min)	5/10	10/10	10/10	10/10	6/7
Breastfeeding Established	No	Yes	No	Yes	No
Ophthalmology					
Abducens paralysis	Yes	Yes	Yes	Yes	Yes
Facial paralysis	Yes	Yes	Yes	Yes	Yes
Previous strabismus surgery	Yes	No	Yes	Yes	No
Lacrimation abnormality	No	No	No	Yes	Yes
Odontology					
DMFS ^a^	0	0	3	1	2
Enamel hypomineralisation ^b^	No	No	Yes	Yes	Yes
HOB, Horizontal overbite (ref. value 3.3 (1.1) mm)	6	4	4	3	1
VOB, Vertical overbite (ref. value 2.7 (1.2) mm)	1	5	4	−4	1
Gingivitis, (range 0–12 index teeth)	4	0	2	5	1
Tongue asymmetry	No	Yes	Yes	No	Yes
NOT-S (range 0–12)	1	1	3	6	8
MOC (ref. mean (range): 45 (25–70) mm) ^c^	n.a.	45	44	53	39
Smile surgery ^d^	No	Yes	No	Yes	No
Craniofacial^e^					
Sagittal intermax. relationsship (ss-n-pg)	n.a.	↑	↑	↑	↑
Maxillary prognathism (s-n-ss)	n.a.	↑	↑	↑	↑
Incisor inclination, superior	n.a.	-	-	↑	-
Alveolar prognathism, superior	n.a.	-	-	-	-
Mandibular prognathism Pg (s-n-pg)	n.a.	-	-	-	-
Incisor inclination, inferior	n.a.	-	-	-	-
Alveolar prognathism, inferior	n.a.	↑	↑	↑	↑
Vertical intermax. relationship (NL/ML)	n.a.	-	-	↑	↑
Maxillary inclination (NSL/NL)	n.a.	-	-	↓	-
Occlusal plane, superior (OLs/NL)	n.a.	↑	-	↓	↑
Mandibular inclination (NSL/ML)	n.a.	-	-	-	↑
Occlusal plane, inferior (OLi/ML)	n.a.	-	↑	↑	↑
Orthopaedics					
Poland syndrome	No	No	No	No	No
Scoliosis	Yes	Yes	Yes	Yes	Yes
Syndactyly	Yes	No	Yes	No	No
Adactyly	Yes	No	Yes	No	No
Camptodactyly	Yes	Yes	Yes	No	No
Bracydactyly	Yes	No	Yes	No	No
CTEV (Club feet)	No	No	No	Yes	No
Pes calcaneovalgus	No	No	Yes	Yes	No
Macrodactyly	No	Yes	Yes	Yes	No
Curly toes	Yes	No	Yes	Yes	Yes
Pedobarography					
Foot length (cm), R/L	n.a.	18.9/19.3	21.4/21.6	21/21	22.2/22.0
Balance eyes-open Distance of Center of Force (cm)	n.a.	92.8	90.4	81.5	123.7
Total foot force (kg), R/L	n.a.	29.9/23.57	n.a./78.8	100.7/83.2	56.1/ n.a.
Total foot contact area (cm^2^) R/L	n.a.	52.39/67.1	n.a./81.2	88.3/81.6	84.4/ n.a.
Total foot peak contact pressure (mmHg), R/L	n.a.	2493/2775	n.a./8586	8080/9470	4331/ n.a.
Region of peak contact pressure, R/L ^f^	n.a.	MH/M3	n.a./ M3	MH/MH	M5/ n.a.

*n.a* Not applicable

^a^number of dental surfaces with decay or filling because of caries. ^b^presence of one or more molars with enamel-hypomineralisation. ^c^maximal opening capacity: interincisal distance on unassisted mouth opening + VOB. ^d^previous transplantation of a muscle grafted from the thigh to the corners of the mouth to improve facial expression [22, 23, 26]. ^e^Craniofacial values compared to mean values and standard-deviation (SD) in 51 untreated healthy females with normal teeth and occlusion. Arrows indicate deviations from reference-value > 1 SD. ^f^MH: Medial Heel Region, M3: Third Metatarsal Region, M5: Fifth Metatarsal Region

### MRI findings

All five patients with MS underwent brain MR imaging. Three had normal scans (patients no. 1-3), one had a mesially placed A-V malformation on the left parietal lobe (patient no. 5) and one had slight enlargement of the ventricular system with hypoplasia of the brainstem and a mild Chiari Type 1 malformation (patient no. 4).

### Facial surgery

**Fig. 2:**
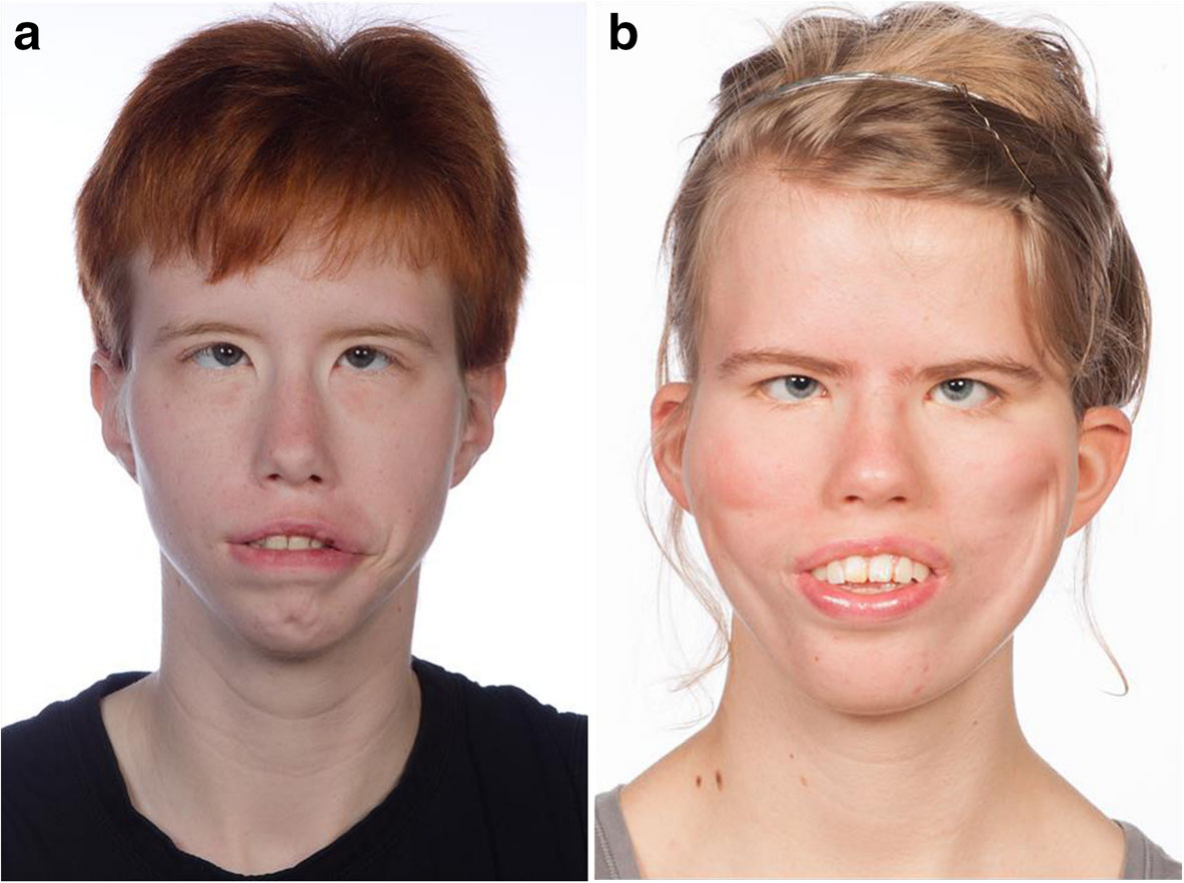
The frontal facial view of two females with Moebius, maximal lip-closure. Patient no. 5 (**a**) have poor facial expression and was only able to force the lower lip into contact with the upper incisors, but the labial surfaces of the upper incisors remained partly uncovered. Patient no. 4 (**b**) have had “smile surgery” performed seen as indentations on the cheeks but was not able to close the lips

**Fig. 3:**
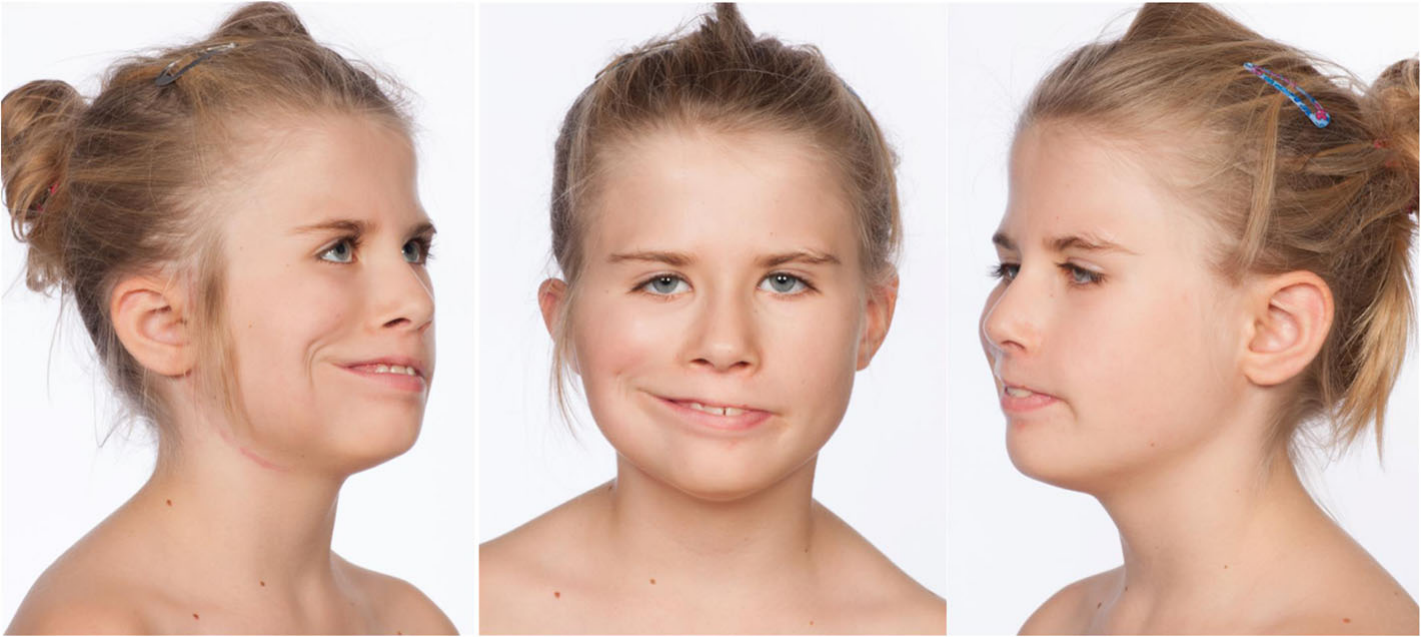
Result of “smile surgery” with frontal facial view and oblique views of patient no 2 when smiling. Patient no. 2 had unilateral (right) “smile surgery” with a free gracilis muscle and nerve transfer. The left side have a minimal function of the lower branches of the left facial nerve

Patients no. 2 and 4 had previously had “smile surgery” – a procedure that improves facial expression through a free gracilis muscle and nerve transfer. Patient no. 4 (Fig. 2) had bilateral surgery and patient no. 2 (Fig. 3) had only unilateral surgery due to a minimal function of the lower branches of the left facial nerve. Patient no. 2 was treated continuously since early childhood with Castillo-Morales’ orofacial therapy [25]. All 5 patients presented with similar dysmorphic features.

### Mental and developmental findings

None of the children with a MS diagnosis were diagnosed with a mental or developmental disorder according to *The International Classification of Diseases* (ICD). Results from *The Strengths and Difficulties Questionnaire* (SDQ) revealed that three out of the five children had a normal SDQ score. One child had a slightly elevated score on “impact of any difficulties on the child’s life” which is not found to indicate any level of risk in the diagnostic predictions. Another child had elevated scores on four items (overall stress, emotional distress, difficulties getting along with other children, and impact of any difficulties on the child’s life). Transformed to diagnostic predictions, the child was considered to have a medium risk for “any disorder” and “emotional disorder” such as anxiety and depression (Table 2).

### Ophthalmological findings

All five patients underwent ophthalmological examination but in some of the patients full assessments were not possible or difficult to obtain. Ophthalmological examination revealed bilateral 6^th^ and 7^th^ nerve palsies in all five patients, verifying the diagnosis of MS. The visual acuity of separate eyes ranged from 0.08 to 1.6. Three of five had previously undergone strabismus surgery (Table 2). All five had lagophthalmus. Two of five patients had abnormal tearing with one patient (patient no. 4) having a lacrimation abnormality known as ‘crocodile tears’ (tearing due to gustatory stimuli) and one patient (patient no. 5) having severely decreased lacrimation and needed treatment. Four patients could cooperate with a corneal examination and had normal corneal sensibility.

### Odontological findings

All five participants underwent dental and orthodontic clinical examination. Patient no 1 could not comply with the cephalometric examination.

The main odontological and cephalometric results are presented in Table 2. Furthermore, agenesis of a maxillary lateral incisor was revealed in one patient (patient no. 3), and in one patient, the dentition was affected by erosion (patient no. 5). The temporomandibular function was assessed in the four girls, only. None had temporomandibular dysfunction. During the NOT-S-interview [23], two patients reported some oral habits (e.g., nail-biting and sucking of lip- or cheek), two patients reported daily drooling, and one patient reported both dryness of the mouth and difficulties in chewing and swallowing. During the NOT-S-examination, three patients had deviations in their face at rest (asymmetry, permanently disclosed lips), and two patients were not able to perform nose-breathing during lip closure. Four patients had poor facial expression (unable to close their eyes tightly, to smile, or to whistle/blow), and three patients had poor orofacial motor function (restricted movements of the tongue, unable to “blow up” their cheeks, or unable to elevate uvula and the soft palate). Only one patient had affected speech according to NOT-S. Except for patient no. 1, none of the patients were able to close their lips. They were able to force the lower lip into contact with the upper incisors, but the labial surfaces of the upper incisors remained partly uncovered by the upper lips (Fig. 2).

**Fig. 4:**
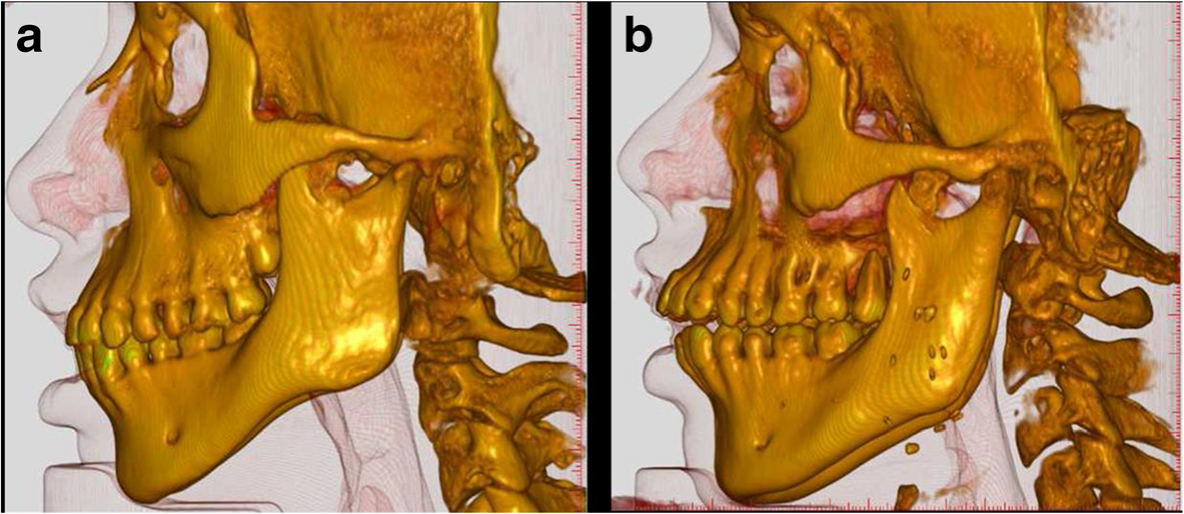
Conebeam CT scanning with 3D-reconstruction of craniofacial structures of two patients with Moebius. Patient no. 5 (**a**) and patient no. 4 (**b**). Both have a large maxillary prognathism in relation to the anterior cranial base (ACB) thus, having relatively retrognathic mandibles. In addition, the mandibular alveolar prognathism in relation to the mandibular base is large. Patient no. 4 (**b**) have severely proclined upper incisors with very divergent jaw-bases opening anteriorly and a marked reduction of the maxillary inclination in relation to the ACB and an anterior open bite

The radiological assessment revealed no craniofacial anomaly and no signs of TMJ deformity. In comparison with reference-values, all cephalometrically assessed patients had a large maxillary prognathism in relation to the anterior cranial base (ACB) thus, having relatively retrognathic mandibles. In addition, the mandibular alveolar prognathism in relation to the mandibular base was large. Two patients had marked divergent jaw-bases (patient no. 4 and 5). In one of them this was caused by a marked reduction of the maxillary inclination in relation to the ACB resulting in an anterior open bite. In the other patient, it was caused by an increased mandibular inclination in relation to ACB but with a normal overbite (Fig. 4, Table 2).

### Orthopaedic findings

**Fig. 5:**
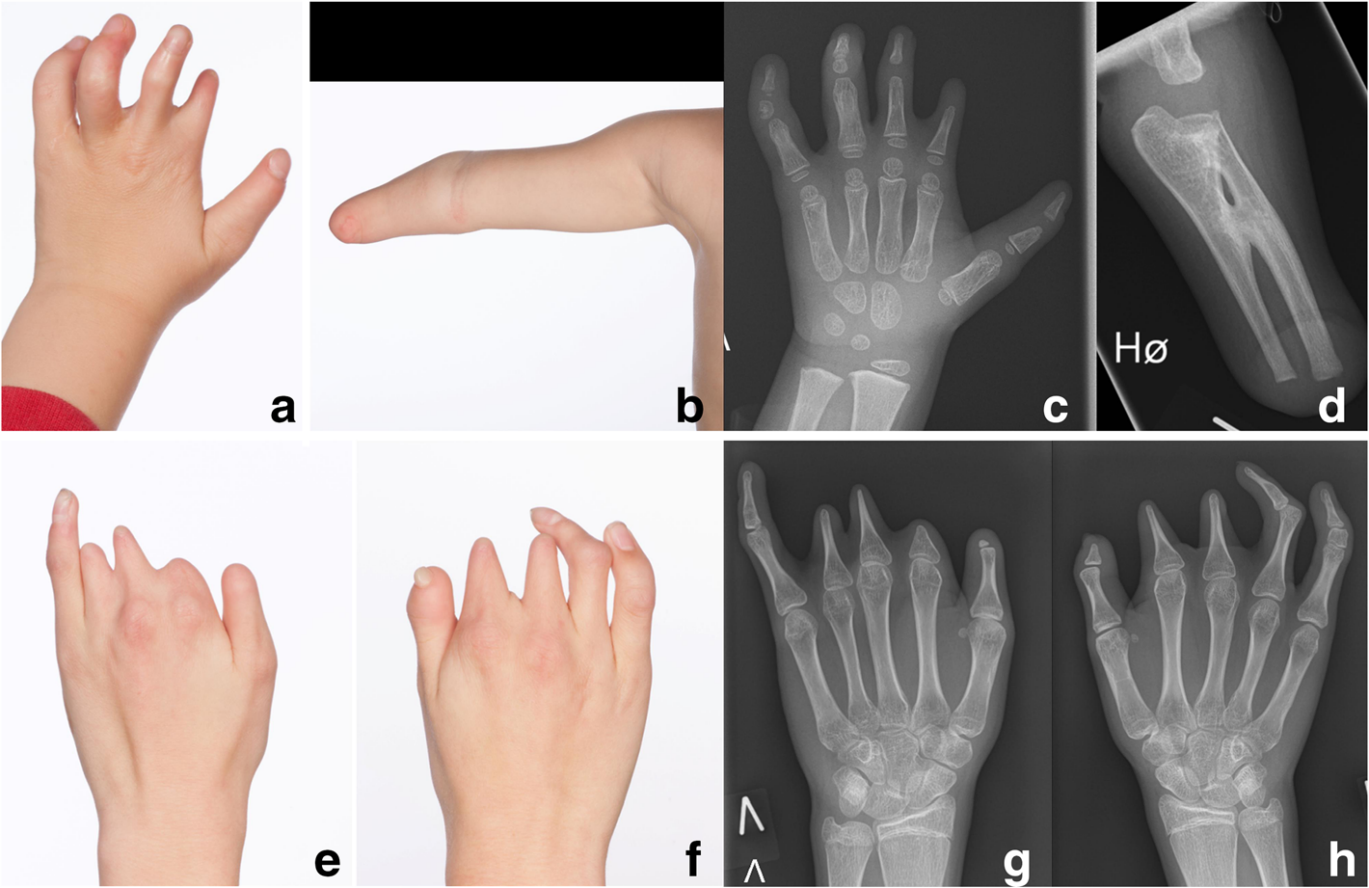
Hands and arm deformities of two patients with Moebius. Clinical photographs and radiographs of the hand and arm of patient no. 1 (**a-d**) shows terminal transverse congenital deficiency of the right forearm with a radioulnar synostosis, brachydactyly (shortness of fingers) of the five fingers on the left hand, slight syndactyly of the left second and third finger and camptodactyly (flexion contracture of the proximal interphalangeal joints) of the left fourth and fifth finger. Clinical photographs and radiographs of the hands of patient no. 3 (**e-h**) shows brachydacyly of all ten fingers, slight syndactyly of the left second and third finger, adactyly of the left second finger and camptodactyly of the right fourth and fifth finger

**Fig. 6:**
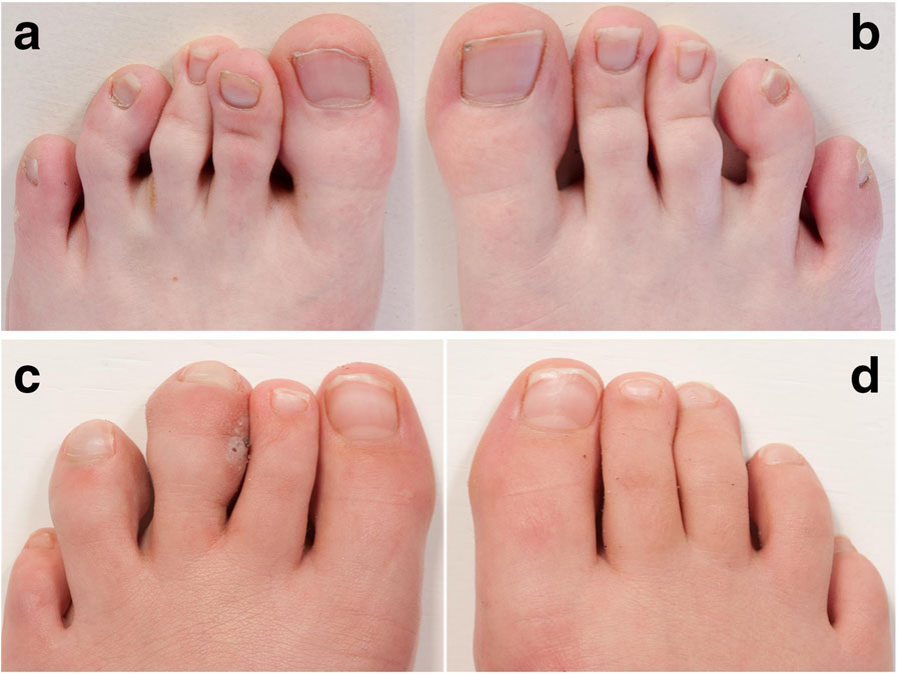
Toe deformities of two patients with Moebius. Clinical photographs of the toes of patient no. 5 (**a-b**) shows curly toes of the left third and fourth toe and the right fourth and fifth toe. Clinical photographs of the toes of patient no. 2 (**c-d**) shows macrodactyly (local gigantism) of the left third and fourth toe

All five patients underwent orthopaedic evaluation. Results are shown in Table 2. All had normal range of motion in elbow, shoulder, hip, knee and ankle and none had genu valgum or vara. Two had minor leg length discrepancy (patients no. 3, 4). Three patients had previous surgery due to foot or hand deformities (patients no. 1, 3, 4). Two of five had extensive upper limb hypoplasia with adactyly, syndactyly and brachydactyly, (Fig. 5). Three patients had camptodactyly. None of the five patients had Poland anomaly and all of the five patients had a mild degree of scoliosis. Two patients had an increased lumbar lordosis (patients no. 2, 4). None of the patients had hip dislocation or dysplasia. One patient had congenital bilateral club-feet. Two of five had calcaneovalgus deformity, and one patient had forefoot adduction (patient no. 2). Three patients had macrodactyly of one or more toes and four patients had curly toes (Fig. 6). One patient had a history of a fracture of the antebrachium after relevant trauma (patient no. 4) and one patient had suffered a lateral patella dislocation (patient no. 3). One patient complained of knee-pain (patient no 5), two patients had observed poor balance (patient no. 2, 5).

**Fig. 7:**
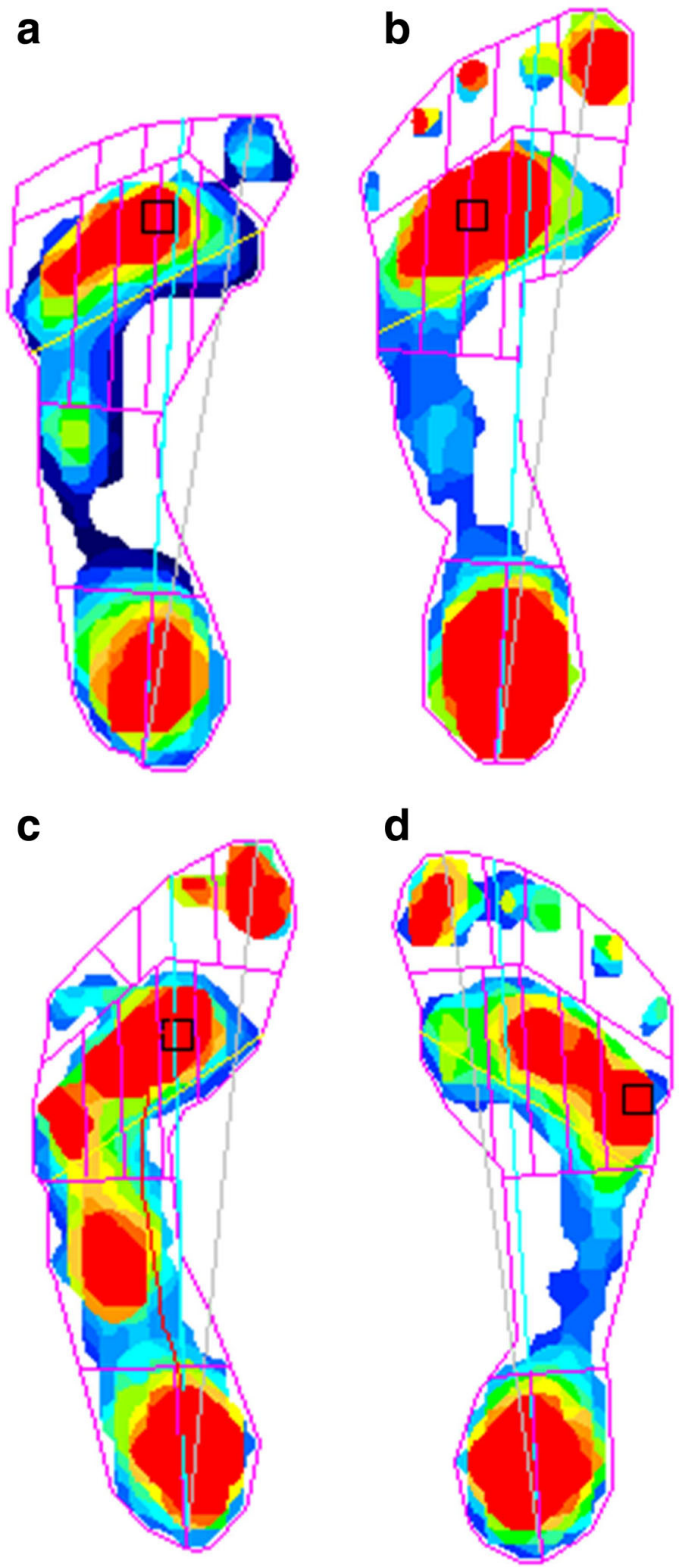
Dynamic pedobarograms of four patients with Moebius. Patient no. 2 (**a**) have forefoot adduction and macrodactyly. The pedobarogram shows adduction of the first toe with the region of peak contact pressure at the third metatarsal head. Patient no. 3 (**b**) have calcaneovalgus position, macrodactyly and curly toes but normal pedobarogram. Patient no. 4 (**c**) have bilateral club feet with previous surgical treatment, calcaneovalgus position, macrodactyly and curly toes. The pedobarogram shows increased pressure in the midfoot area with a total foot peak contact pressure of 8080 mmHg. Patient no. 5 (**d**) have calcaneovalgus position and curly toes but normal pedobarogram

Pedobarography showed plantar pressures within normal ranges both totally and in the sub regions of the foot in both static and dynamic tests (Fig. 7, Table 2). All four, undergoing pedobarography, range between normal and poor balance when standing both with eyes open and closed. The patient with the measured worst balance also had a self-reported balance problem (patient no. 5).

## Discussion

In the present study we have set up diagnostic criteria and examined all patients with MS or a Moebius-like diagnosis. Only half of the examined patients could maintain the diagnosis of MS. This adheres to a recent study by MacKinnon et al. [2], who found that 19% of the enrolled patients did not meet the minimum diagnostic criteria (MDC) which they defined as ‘a congenital, uni- or bilateral, nonprogressive facial weakness and limited abduction of the eye(s) and full vertical motility’. Opposite, the diagnostic criteria for present study were defined as *bilateral*, congenital facial and abducens paralysis, which even further reduces the number of patients maintaining the diagnosis of MS.

The advantage of the present study is the multidisciplinary approach taken, which gives a thorough analysis of all symptoms in the evaluated group of patients with validated MS. An immense range of symptoms have been reported relating to MS but only a few are diagnostically relevant. Bilateral facial and abducens paralysis are of importance since accurate diagnosis is the basis for correct discussion of other relevant concomitant symptoms of MS, genetic testing and evaluation of prognosis. Therefore, the present study proposes more stringent diagnostic criteria for MS, which increases the potential that MS is the correct diagnosis. Some case reports on patients with MS are, in our opinion, not reports of classical MS, but more likely a part of the heterogenic group of syndromes called “Oro-Mandibular-Limb hypogenesis syndrome” (OMLH) characterised by varying cranial nerve palsies and craniofacial anomalies and limb anomalies sometimes combined with aplasia of the pectoral muscle [6]. These cases should only be compared to classical patients with MS with caution since the symptomatology; phenotype and cause might be a completely different entity. This is in accordance to Miller et al. [7], who state that due to differences of opinions regarding findings required to make the diagnosis of MS evaluations of cases reported in the literature is sometimes difficult. In one of the five patients not meeting the diagnostic criteria the genetic testing revealed a TUBB3 mutation, causing congenital fibrosis of the extraocular muscles type 3, which is an alternative diagnosis also found in other studies [2].

As opposed to both Briegel et al. [5] who have found an equal sex-ratio of 1:1 in MS and Strömland et al. [6] who have found a ratio of 1:3, favouring males, this study finds a sex-ratio of 4:1 favouring females. Due to the low number of patients in all of these studies no consensus regarding the true sex-ratio can be determined.

The causality of MS is not adequately investigated, but the present study may indicate a connection between uterine abnormalities and the cascade of secondary events in embryonic development. Two of the five patients in present study were born to mothers with uterine abnormalities (unicornuate and bicornuate uterus), which may be suspected of being a cause to the damages to the foetus due to either hemorrhage or ischemia at a critical period in the embryonic development of structures including the abducens and facial nerve. Furthermore two of five patients had an inappropriate intrauterine position which may be due to uterine abnormalities. Strömland et al. [6] found adverse pregnancy events in 16 of 25. Three of five patients in the present study had problems establishing breastfeeding in the neonatal period and had additional problems in the feeding process with swallowing and chewing. Recently, de novo mutations have been reported to cause MS indicating a genetic aetiology of MS, which further increases the possibility for a correct diagnosis of MS [8]. However, studies still need to investigate the relationship between genotype, phenotype and previous reported symptomatology in order to set up guidelines for distinguishing classical MS from other syndromes included in the group of OMLH syndromes. Furthermore, studies need to investigate whether a genetic predisposition followed by a vascular cascade of secondary events and uterine abnormalities might both be crucial factors in the development of classical bilateral MS.

As stated by Moralez-Cháves et al. [11] facial disability, including smiling, might be a disabling condition both functionally, psychologically and aesthetically. Children with MS are described with facial blankness and inability to express emotions and are hence described as “Children without a smile”. Functions of the facial muscles are essential for both verbal and non-verbal communication as well as social interaction. In the present study, the two patients with the highest SDQ scores on “emotional stress” and “overall stress” also had the highest NOT-S score. The high NOT-S score is an indication of a poor oral motor function, which includes facial disability. Thus, an association between poor oral motor function and high emotional and overall stress may exist. However, the two patients with high scores were also the patients with the highest age. Presumably, age also influences the impact of facial disability on the patient’s emotional and overall stress in society. Of the two patients who had smile surgery performed, patients no. 4 had high SDQ and NOT-S scores but patient no. 2 had a relatively good oral motor function with only minor facial disability (low NOT-S score) and low emotional and overall stress. This adds to the impression of an association between the facial disability and both emotional and overall stress. However, the limited sample size allows no conclusive statement on the potential association.

Although minor deviations were seen in relation to the dentition (agenesis and hypomineralisation) no consistent abnormality could be associated with MS, and the teeth were healthy. This is in contrast to previous reports of dental agenesis [1, 15] and the reported risk of rampant caries [16]. The NOT-S revealed an affection of the orofacial motor function in all patients. That finding is expected in a disease with facial palsy and is in accordance with other studies [1, 6, 16].

Previous studies have reported severe mandibular retrognathism in patients with MS [6, 16]; in contrast, the present study found a craniofacial and dentofacial morphology dominated by a maxillary prognathism combined with a normal mandible causing an intermaxillary discrepancy. A variance of dentoalveolar compensations or dysplastic remodelling was seen. Severe malocclusion only played a minor role.

This study finds macrodactyly in three of five patients, curly toes in four of five patients and a mild scoliosis in all five. These findings have not previously been associated with MS. In addition, this study has not found any MS patients with Poland anomaly, opposite to the findings of other studies.

## Conclusions

MS is an extremely rare disease with a risk of misdiagnosis due to differing diagnostic criteria. The present study proposes congenital bilateral paralysis of the abducens and facial nerves as standard diagnostic criteria for MS. In patients where these criteria are not met the diagnosis may be a Moebius-like syndrome or Oro-Mandibular-Limb hypogenesis syndrome (OMLH).

No definitive conclusion on causality or new symptomatology can be made due a low patient number, although some trends can be appreciated.

## Abbreviations

ACB: Anterior cranial base (ACB)
CPAP: Continuous positive airway pressure
DMFS: Decayed missed or filled surfaces due to caries
HOB: Horizontal overbite
ICD: The International Classification of Diseases
MDC: Minimum diagnostic criteria
MOC: Mouth opening capacity
MS: Moebius sequence
NOT-S: The Nordic Orofacial Test- Screening
OMLH: Oro-Mandibular-Limb hypogenesis syndrome
SDQ: The Strengths and Difficulties Questionnaire
TMJ: Temporo-mandibular joint
VOB: Vertical overbite

## References

[1] Sjögreen L, Andersson-Norinder J, Jacobsson C (2001). Development of speech, feeding, eating, and facial expression in Möbius sequence. Int J Pediatr Otorhinolaryngol.

[2] MacKinnon S, Oystreck DT, Andrews C, Chan WM, Hunter DG, Engle EC (2014). Diagnostic distinctions and genetic analysis of patients diagnosed with Moebius syndrome. Ophthalmology.

[3] Grazadio C, Lorenzen MB, Rosa RFM, Pinto LLC, Zen PRG, Travi GM (2010). New report of a familial case of Moebius syndrome presenting skeletal findings. Am J Med Genet Part A.

[4] Briegel W, Schimek M, Kamp-Becker I, Hofmann C, Schwab KO (2009). Autism spectrum disorders in children and adolescents with Moebius sequence. Eur Child Adolesc Psychiatry.

[5] Briegel W (2006). Neuropsychiatric findings of Möbius sequence - a review. Clin Genet.

[6] Strömland K, Sjögreen L, Miller M, Gillberg C, Wentz E, Johansson M (2002). Möbius sequence − a Swedish multidiscipline study. Eur J Paediatr Neurol.

[7] Miller MT, Stömland K (1999). The Möbius sequence: a relook. J AAPOS.

[8] Tomas-Roca L, Tsaalbi-Shtylik A, Jansen JG, Singh MK, Epstein JA, Altunoglu U (2015). De novo mutations in PLXND1 and REV3L cause Möbius syndrome. Nat Commun.

[9] Dufke A, Riethmüller J, Enders H (2004). Severe congenital myopathy with Möbius, Robin, and Poland sequences: new aspects of the Carey-Fineman-Ziter syndrome. Am J Med Genet.

[10] Verloes A, Bitoun P, Heuskin A, Amrom D, Van de Broeck H, Nikkel SM (2004). Möbius sequence, Robin complex, and hypotonia: severe expression of brainstem disruption spectrum versus Carey-Fineman-Ziter syndrome. Am J Med Genet.

[11] Morales-Chávez M, Ortiz-Rincones MA, Suárez-Gorrin F (2013). Surgical techniques for smile restoration in patients with Möbius syndrome. J Clin Exp Dent.

[12] Lima LM, Diniz MB, dos Santos-Pinto L (2009). Möbius syndrome: clinical manifestations in a pediatric patient. Pediatr Dentist.

[13] Kremer H, Kuyt LP, van den Helm B, van Reen M, Leunissen JAM, Hamel BCJ (1996). Localization of a gene for Möbius syndrome to chromosome 3q by linkage analysis in a Dutch family. Hum Mol Genet.

[14] Verzijl HT, van den Helm B, Veldman B, Hamel BC, Kuyt LP, Padberg GW, Kremer H (1999). A second gene for autosomal dominant Möbius syndrome is localized to chromosome 10q, in a Dutch family. Am J Hum Genet.

[15] Domingos AC, Lopes SLCP, Almeida SM, Boscolo FN, Whaites EJ (2004). Poland-Moebius syndrome: a case with oral anomalies. Oral Dis.

[16] Magalhaes M, Araújo L, Chiaradia C, Fraige A, Zamunaro M, Mantesso A (2006). Early dental management of patients with Mobius syndrome. Oral Dis.

[17] Turk AE, McCarthy JG, Nichter LS, Thorne CH (1999). Moebius syndrome: the new finding of hypertrophy of the coronoid process. J Craniofac Surg.

[18] Verzijl HTFM, van Es N, Berger HJC, Padberg GW, van Spaendonck KPM (2005). Cognitive evaluation in adult patients with Möbius syndrome. J Neurol.

[19] Bandim JM, Ventura LO, Miller MT, Almeida HC, Costa AES (2003). Autism and Möbius sequence: an exploratory study of children in northeastern Brazil. Arq Neuropsiquiatr.

[20] Johansson M, Wentz E, Fernell E, Strömland K, Miller MT, Gillberg C (2001). Autistic spectrum disorders in Möbius sequence: a comprehensive study of 25 individuals. Dev Med Child Neurol.

[21] Goodman R (2001). Psychometric properties of the strengths and difficulties questionnaire. J Am Acad Child Adoleso Psychiatry.

[22] Muller L, van Waes H, Langerweger C, Molinari L, Saurenmann R (2013). Maximal mouth opening capacity: percentiles for healthy children 4-17years of age. Pediatr Rheumatol.

[23] Bakke M, Bergendal B, Sjögreen L, Asten P (2007). Development and evaluation of a comprehensive screening for orofacial dysfunction. Swed Dent J.

[24] Solow B (1966). The pattern of craniofacial associations.

[25] Limbrock GJ, Fischer-Brandies H, Avalle C (1991). Castillo-Morales’ orofacial therapy: treatment of 67 children with Down Syndrome. Dev Med Child Neurol.

[26] Ingerslev CH, Solow B (1975). Sex differences in craniofacial morphology. Acta Odontol Scand.

